# The anisotropic field of ensemble coding

**DOI:** 10.1038/s41598-021-87620-1

**Published:** 2021-04-15

**Authors:** David Pascucci, Nadia Ruethemann, Gijs Plomp

**Affiliations:** 1 Department of Psychology, University of Fribourg, Fribourg, Switzerland; 2 Laboratory of Psychophysics, Ecole Polytechnique Fédérale de Lausanne (EPFL), Lausanne, Switzerland

**Keywords:** Human behaviour, Perception

## Abstract

Human observers can accurately estimate statistical summaries from an ensemble of multiple stimuli, including the average size, hue, and direction of motion. The efficiency and speed with which statistical summaries are extracted suggest an automatic mechanism of ensemble coding that operates beyond the capacity limits of attention and memory. However, the extent to which ensemble coding reflects a truly parallel and holistic mode of processing or a non-uniform and biased integration of multiple items is still under debate. In the present work, we used a technique, based on a Spatial Weighted Average Model (SWM), to recover the spatial profile of weights with which individual stimuli contribute to the estimated average during mean size adjustment tasks. In a series of experiments, we derived two-dimensional SWM maps for ensembles presented at different retinal locations, with different degrees of dispersion and under different attentional demands. Our findings revealed strong spatial anisotropies and leftward biases in ensemble coding that were organized in retinotopic reference frames and persisted under attentional manipulations. These results demonstrate an anisotropic spatial contribution to ensemble coding that could be mediated by the differential activation of the two hemispheres during spatial processing and scene encoding.

## Introduction

Humans can accurately estimate the statistical properties of an ensemble of stimuli at a single glance^1^. The average ripeness of a batch of apples, the direction of motion of a dense flock of birds, and the emotional expression of a whole crowd of people, are all estimates that require little effort and can be made within a fraction of a second. Representing ensembles by their statistical properties, or *ensemble coding* (EC^2,3^), is an efficient way to optimize the processing of complex and redundant information, allowing for a coarse initial ‘gist’ of the scene^4,5^.

In EC, a few milliseconds of presentation time are enough to estimate a variety of statistical features, including the average location of an ensemble of stimuli^6^, their centroid^7^, direction of motion^8^, mean size and orientation^1,9,10^, as well as higher-level aspects such as the average emotion of a group of human faces^11^. For basic visual features like orientation and motion, EC can be explained by the simple pooling of low-level signals across populations of receptors sensitive to orientation and motion^2,10^. For other features, particularly for size, the underlying mechanisms are less straightforward, in part because there are no known size receptors in the brain^12,13^. Nevertheless, research on EC of size has shown that humans can reliably estimate the mean size of an ensemble of stimuli independently of their duration^14^ and set size^1,14,15^ (but see^16^), even when attention is focused elsewhere^7,17^ or when conscious access to part of the ensemble is restricted^18^. This suggests that size averaging occurs automatically and in parallel across the visual field, bypassing the capacity limits of our attentional system^19^. Such an automatic and parallel mode of processing is generally assumed to operate in an *holistic* (i.e., all items in a group contribute equally to the estimated mean size) and *isotropic* fashion (i.e., all items contribute equally, independently of their position in the visual field).

Whether size averaging, and EC in general, is supported by truly holistic and isotropic modes of processing remains unclear. Several studies, for instance, indicate that stimuli may be weighted differently depending on their location in the visual field^20^, their saliency^21^ and their distance to the group mean^22^. Investigating the presence of systematic biases, asymmetries, and anisotropies in EC is important, because such knowledge can be used to understand the underlying mechanisms. Indeed, perception and attention operate non-uniformly in space at many stages of processing. For example, anisotropies and eccentricity effects may arise early in the processing stream, due to the functional organization of the visual system itself, with a higher spatial resolution at the fovea^23^ and asymmetries in the processing of object and spatial information between the upper and lower visual field^24^. Also, eye movements during picture scanning^25^, as well as performance in attentional, perceptual, and memory tasks^26–29^ are affected by systematic left-side biases, likely due to the right-hemisphere dominance in spatial orienting^30,31^. A typical example is the pseudo-neglect effect, in which healthy subjects show a leftward bias in the perception of basic visual features, such as line segments, numerosity, and brightness^32^. Hemispheric asymmetries also exist in the perception of global and local features, with an advantage for representing global features in the left visual field (right hemisphere) and local details in the right visual field (left hemisphere)^33,34^. Despite the ubiquity of these biases in perception and attention, no systematic investigation in the context of EC exists.

In this work, we use the Spatial Weighted Average model^35^, to derive the contributions of different parts of the visual field to the reported average size of an ensemble. Typically, previous studies have used forced-choice and adjustment tasks to compare the estimated mean of an ensemble against the true mean^1,3^. Here, we use trial-by-trial variations in the individual size of an array of disks, along with their position in the visual field, to estimate two-dimensional maps (spatial weighting maps; SWMs). SWMs describe the distribution of weights with which local sensory signals contribute to the estimated summary statistic. In three experiments, we evaluate the presence of anisotropic fields in EC of size while presenting ensembles of disks with different degrees of dispersion, at different retinal locations, and under different attentional demands. Our results reveal highly anisotropic SWMs, that are centered in a retinotopic reference frame, and resemble well-known horizontal asymmetries and left-side biases in spatial processing. Interestingly, the observed anisotropies were only gated, but not prevented by an explicit manipulation of spatial attention. We speculate that EC of size could be mediated by the unbalanced encoding of global vs. local information between the two hemispheres.

## Results

### Experiment 1

**Figure 1:**
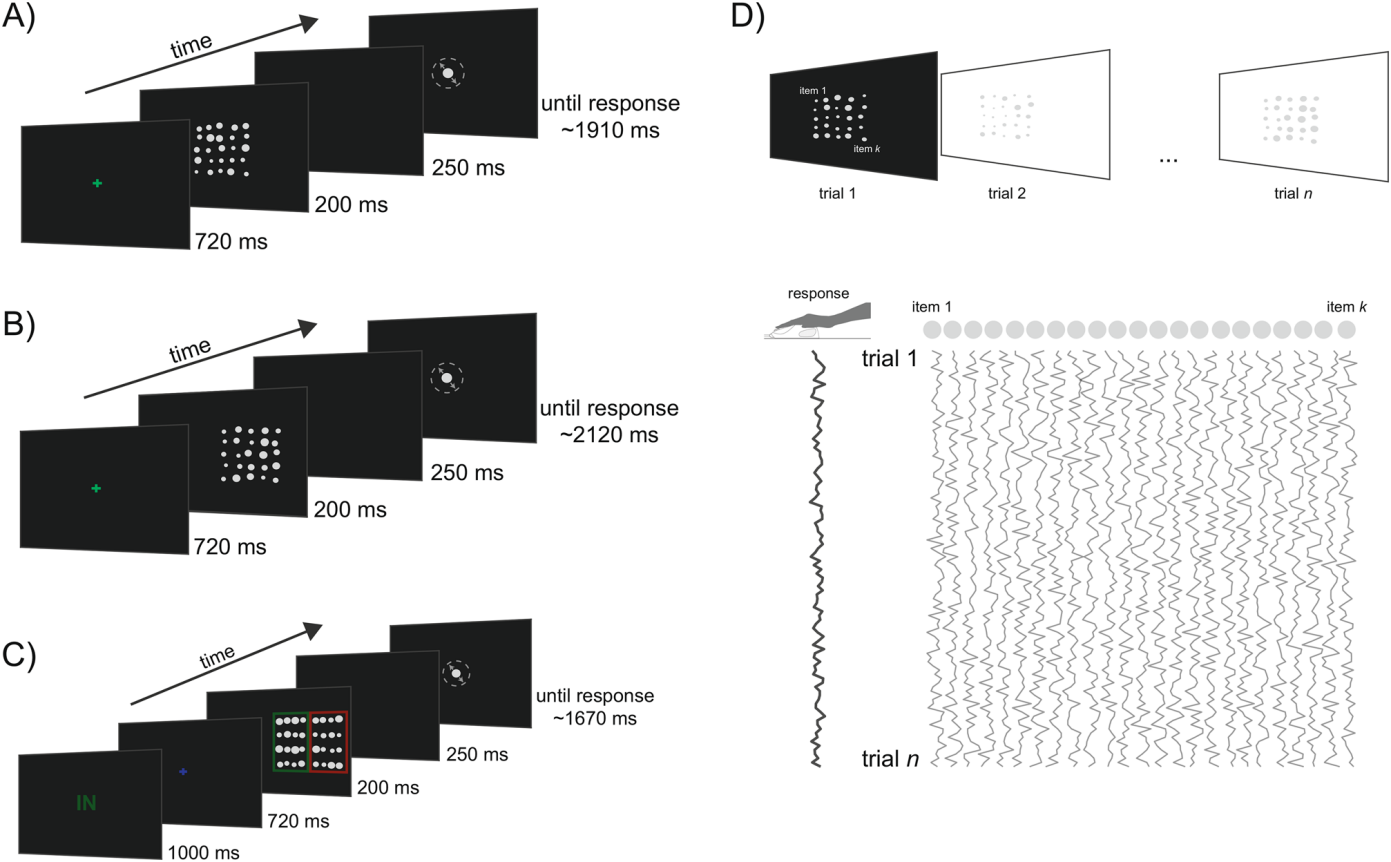
Sequence of events in the ensemble coding tasks. (**A**) Example of one trial in Experiment 1. Stimuli were presented at the fovea with different degrees of dispersion (see “General methods” section). After the fixation cross, an ensemble of 25 disks was presented for 200 ms, followed by a blank interval. The task was to adjust the size (diameter) of a single central disk so that it reproduced the average size of the set of stimuli in the ensemble. (**B**) Example of one trial in Experiment 2, with the ensemble presented on the right side of the display. In this experiment, ensembles could randomly appear on the left, in the center or on the right side. (**C**) Example trial in Experiment 3. At the beginning of each trial, participants received a visual cue (here ‘IN’) indicating the region of the screen where the relevant ensemble was presented (here the internal region). After a fixation interval, two ensembles separated by colored frames were presented. Participants had to reproduce the average size of stimuli inside the relevant ensemble, indicated by a frame that matched the color of the initial cue. (**D**) Derivation of spatial weighting maps (SWMs). Local variations in the size of each disk across trials were used as multivariate regressors explaining the trial sequence of adjustment responses. Two-dimensional SWM were obtained by estimating the weight (regression coefficient) of each disk at each location on participants’ responses.

**Figure 2:**
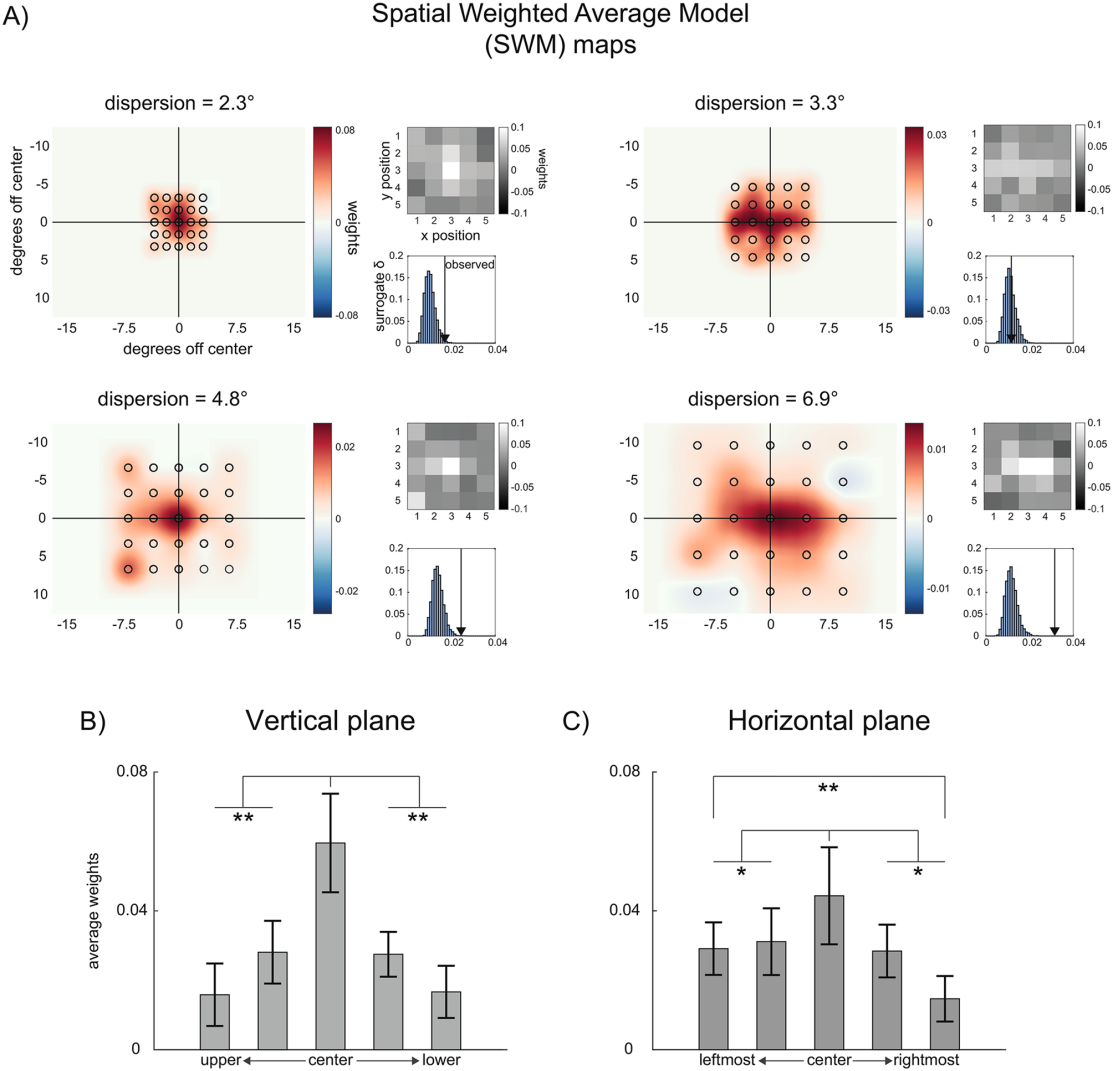
Results of Experiment 1. (**A**) SWM maps for each dispersion condition. Grey-scale maps visualize the original weights in matrix coordinates, estimated through the spatial weighted averaging model. Red and blue maps are the corresponding smoothed heatmaps, in screen coordinates. Histograms show the results of the permutation statistic, comparing the observed distance of weights ($\delta$) from a uniform distribution (black arrow) to that of randomly permuted surrogate maps (blue bars). (**B**) Comparison of weights along the vertical plane, collapsing columns of SWM maps. (**C**) Comparison of weights along the horizontal plane, collapsing rows of SWM maps. Error bars correspond to the standard deviation of the mean. * = *p* < 0.05; ** = *p* < 0.001.

In a first experiment, we investigated whether participants give equal weight to all the items of an ensemble, independently of their location and dispersion in the visual field. To this aim, we derived SWM maps (see Fig. 1 and “General methods” section) in four conditions in which participants reported the average size (diameter) of an ensemble of twenty-five disks presented at the center of the screen with four levels of dispersion (see Fig. 1A). Figure 2 shows the SWM maps quantifying the weight assigned to each disk depending on its location and dispersion in the visual field (Fig. 2A). The distribution of weights within ensembles deviated significantly from a theoretical uniform distribution, as evident in three out of four dispersion levels (all *p* < 0.01, except for dispersion = 3.33°, *p* = 0.37; permutation statistics).

To test whether the observed anisotropies were statistically different across dispersion levels, we compared the distribution of weights in the four conditions using the permutation F-statistic (see “General methods” section). This analysis revealed no significant difference in the distribution of weights across dispersion levels (all *p* > 0.05). Thus, the presence of systematic spatial anisotropies was independent of the dispersion of disks in the visual field. To further characterize the nature of these biases, we collapsed across dispersion levels and compared weights assigned to disks along the vertical and horizontal plane. This revealed a bias toward the center and left-hand side of the ensemble (center vs. lower and upper locations: *t*(31) = 4.17, *p* < 0.001, *d*′ = 0.74, Fig. 2B; center vs. left-side and right-side locations: *t*(31) = 2.12, *p* = 0.04, *d*′ = 0.37; leftmost column vs. rightmost column: *t*(31) = 3.25, *p* = 0.003, *d*′ = 0.57; all other *p* > 0.05; paired t-tests, Fig. 2C).

Experiment 1 indicated that during size averaging, human subjects tend to assign higher weights to the subset of items in the central-left portion of the ensemble, revealing an anisotropic and non-uniform spatial averaging field. This is in line with the hypothesis that EC operates through the preferential weighting of certain items over others in the group^21^ and confirms previous reports of a left-side bias^20^. In our experiment, however, ensembles were always presented at the center. This does not allow us to clarify whether the non-uniform weighting was due to a bias in a retinotopic reference frame, which may support anisotropies at the early stages of encoding, or to a bias in an object-based reference frame, which may support later stages (e.g., a bias to direct attention toward the center of an ensemble^36^). To disentangle these two possibilities, we performed a second experiment in which ensembles were presented at three different spatial locations, randomly intermixed across trials (Fig. 1B).

### Experiment 2

**Figure 3:**
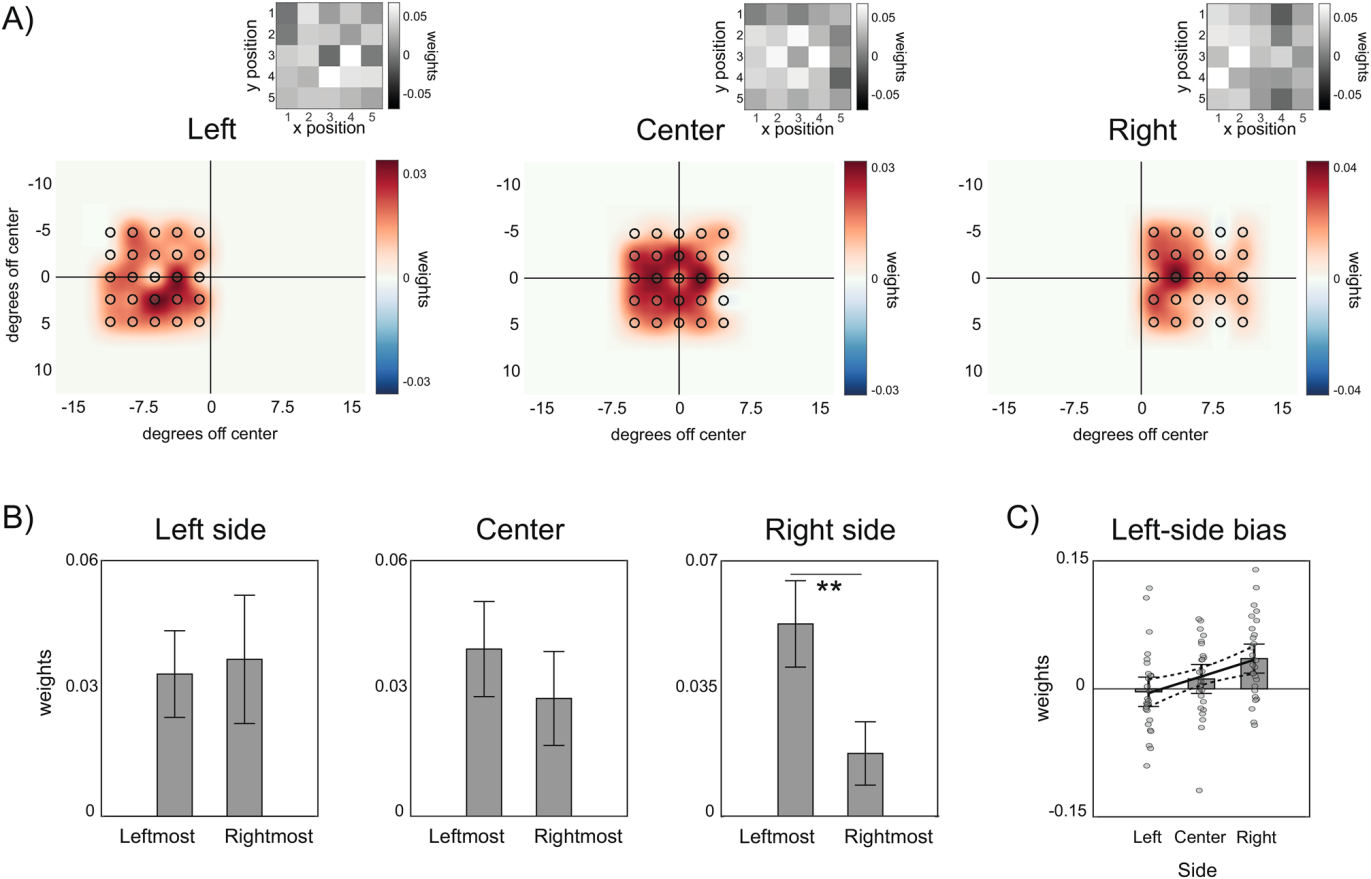
Results of Experiment 2. (**A**) SWM maps for ensembles presented on the left side, center and right side of the screen. (**B**) Comparison of weights in the two leftmost and two rightmost columns of each condition, collapsing across the rows of the SWM maps. (**C**) Linear regression on the leftward bias (weights on two leftmost columns minus weights on two rightmost columns) as a function of the ensemble location, showing the increase of the bias from left to right.

In Experiment 2, we presented ensembles of twenty-five disks at three locations randomly determined on each trial (left: − 6°, center: 0° and right: 6° off fixation). In line with Experiment 1, two out of three maps presented significant anisotropies (all *p* < 0.05, except for Center, *p* = 0.07; permutation statistics). However, as Fig. 3A shows, maps obtained for ensembles presented in the left and right side of the visual field had a qualitatively different distribution of weights compared to Experiment 1. For peripheral locations, larger weights were no longer distributed around the center of the ensemble but toward the side that was closer to the center of the visual field.

To directly test the presence of a leftward bias, we compared the averaged weights in the two leftmost columns of possible positions with those in the two rightmost columns, for each location separately. This comparison revealed a bias toward the left when ensembles were presented in the right visual field (Fig. 3B; leftmost columns minus rightmost columns: *t*(26) = 3.89, *p* < 0.001, *d*′ = 0.75; *p* > 0.05 for all other comparisons). The leftward bias followed a linear trend over ensembles location, increasing from the left to the right side of the visual field (Fig. 3C; linear slope over ensembles location: 0.019 ± 0.006, *p* = 0.002). This result confirmed a spatial bias toward the center-left of the visual field, organized in retinotopic coordinates and increasing as the items of the ensemble fall outside the preferential zone for averaging. An alternative possibility, however, is that participants may still have systematically paid more attention to the center of the screen since the exact location of the ensemble in each trial was unknown in advance. This, in turn, could have promoted a bias toward stimuli within the central focus of attention. Although this possibility does not fully account for the leftward bias, we performed a final experiment to evaluate the role of spatial attention in more detail, and to validate our findings.

### Experiment 3

Our first two experiments revealed a systematic center-left bias in the distribution of weights during size averaging. One possibility is that participants maintained their focus of attention at the center of the display in both experiments. This, in turn, could have contributed to the differential weighting of items falling inside or outside the focus of attention. In this final experiment, therefore, we addressed the role of spatial attention more directly, by evaluating whether the observed anisotropies can be modulated by an explicit manipulation of spatial attention. Participants performed a modified version of the size averaging task in which two ensembles (16 disks each), grouped by two colored frames (see “General methods” section and Fig. 1C), were presented at the same time. The two ensembles could appear either on the left or right side of fixation. At the beginning of each trial, a written cue instructed participants to attend only to the ensemble appearing on the internal or external part of the display while ignoring the other. The task was to report the average size of the disks in the attended ensemble. In this way, we were able to distinguish between three distinct hypotheses: (1) that the anisotropies originate from an involuntary and automatic bias in EC that cannot be prevented (i.e., items in the center-left region are given higher weights, even when task-irrelevant); (2) that the anisotropies reflect biases in how visual spatial attention is directed in the absence of specific instructions and thus should disappear in the present task; (3) that the anisotropies emerge early at the encoding stage but can be modulated by spatial attention (i.e., attention determines the region of the visual field that is selected for averaging, but within that region, center-left anisotropies persist).

**Figure 4:**
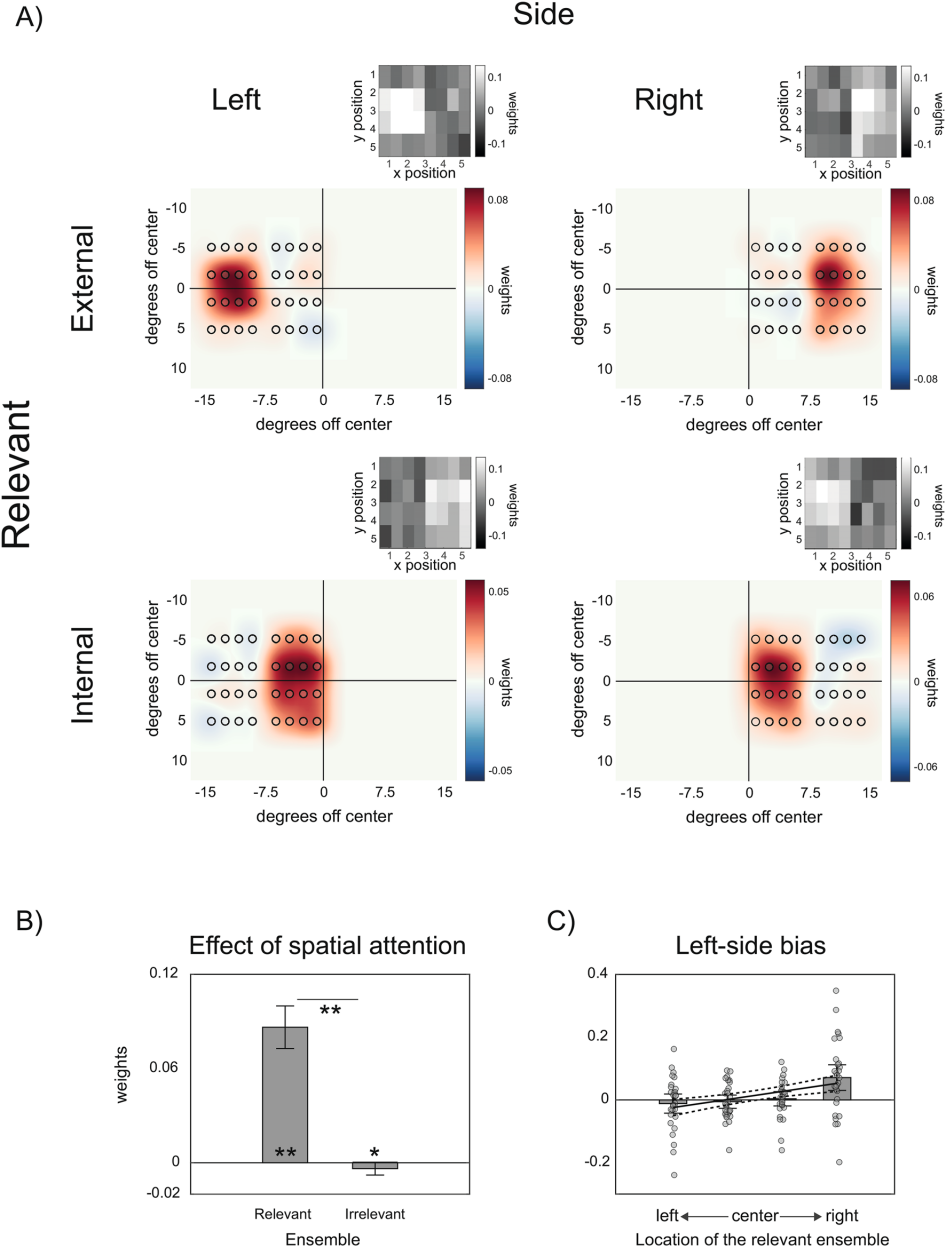
Results of Experiment 3. (**A**) SWM maps for the four combinations of ensemble location (left and right) and relative position of the cued (relevant) ensemble (internal and external). (**B**) Overall weights assigned to stimuli in the relevant and irrelevant ensemble. (**C**) Linear regression on the leftward bias as a function of the actual location of the relevant ensemble.

Figure 4 shows the SWM obtained for each of the four conditions in Experiment 3, depending on whether the ensembles were presented to the left or right of fixation and whether participants attended the external or internal part of the display. As evident from these maps, participants selected and averaged only the size of the disks within the cued ensemble, with a large difference between the overall weights assigned to attended and non-attended disks (*t*(28) = 10.95, *p* < 0.001, *d*′ = 2.03, Fig. 4B). Moreover, weights assigned to non-attended disks were on average negative (*t*(28) = − 1.93, *p* = 0.031, *d*′ = 0.36, one-tailed t-test against zero). Crucially, even if our manipulation of spatial attention strongly determined the overall distribution of weights in the visual field, the left-side bias was still present, following a linear trend over the location of the attended ensemble, from the external left-hand side to the external right-hand side of the visual field (Fig. 4C, linear slope over ensembles location: 0.025 ± 0.007, *p* < 0.001). This result supported our third hypothesis, that spatial attention can gate, but not eliminate, the observed anisotropies in EC.

## Discussion

In the present study, we used the Spatial Weighted Averaging models to investigate the contribution of individual stimuli during ensemble coding. By varying the dispersion, eccentricity, and relevance of an ensemble of stimuli, we demonstrated that EC operates in a strongly anisotropic and non-holistic field, with a larger contribution of stimuli in the center-left portion of the visual field. This anisotropic field was organized in a retinotopic coordinate frame and persisted under explicit manipulations of spatial attention.

Our results challenge the notion of a holistic and uniform mode of statistical processing^15,37^, by revealing that individual stimuli do not equally contribute to the mean. There is evidence in the literature of a preferential weighting in EC. For instance, it has been shown that salient stimuli contribute more to the mean^21^ whereas outlier stimuli contribute less^22,38^. Our work demonstrates that the retinal location is another important factor, independent of the salience and distributional properties of individual stimuli: EC operates in an anisotropic spatial field.

The anisotropic field of EC closely resembles well-known central and leftward biases in attention, perception, and viewing behavior^25–27,29^. Typically, leftward biases find several explanations, the most obvious one being reading scanning habits^39,40^. There is reasonable evidence, however, to exclude the possibility that scanning habits are the only factor involved. Leftward biases are indeed also present in right-to-left readers^41^, they become evident early in the development, and they are not limited to humans and primates^42,43^.

A widely accepted alternative hypothesis is that leftward biases arise from brain asymmetries in the control of spatial attention^30,44^. In line with this view, recent work by Li and Yeh (2017) indicated a leftward bias in EC that was modulated by spatial attention^20^. In their study, mean size judgments were affected by whether the mean size on the left was larger or smaller than the mean size on the right. They found that the leftward bias disappeared when attention was pre-cued toward the right and was even reversed when items on the right were presented earlier. This led the authors to conclude that EC is affected by a bias in the automatic deployment of spatial attention, which causes the prior entry of stimuli on the left side^20^. In our Experiment 3, we found that diverting attention from the center cannot entirely prevent the bias. We pre-cued participants' attention toward either the external or the internal regions of the visual field and we observed that, within the attended regions, the leftward bias was still present (Fig. 4). This does not exclude the possibility that the bias can be counteracted by experimentally inducing attentional shifts toward the right, as in Li and Yeh^20^. However, it shows that central and leftward biases are not simply due to the way in which attention is automatically deployed in space, in the absence of specific instructions and cues.

The results of Experiment 3 are also important for the role of attentional selection in EC. Previous work suggests that the inclusion of irrelevant stimuli in summary statistics cannot be prevented^15,45^. For instance, in a study where participants estimated the mean length of an ensemble of lines, with task-relevant stimuli (e.g., horizontal lines) spatially intermixed with irrelevant ones (e.g., vertical lines), Oriet and Brand^45^ found that mean length judgments were systematically affected by the length of irrelevant lines. Our results, instead, demonstrate that relevant and irrelevant stimuli, with identical features and in close proximity, can be efficiently segregated into distinct sets, based on their location. Thus, when relevant and irrelevant stimuli are not spatially intermixed, EC can be highly selective and exclusive.

An interesting and somewhat surprising finding is that the estimated mean size was slightly repelled away from the average size of the irrelevant ensemble (Fig. 4B). This suggests that the segregation of stimuli into distinct sets, and the encoding of their respective properties, occurs prior to attentional selection. The presence of repulsive biases may be evidence of a relational format in EC: in representing an ensemble’s mean, its difference from the mean of other ensembles in the scene is exaggerated. This possibility requires experimental investigation.

Taken together, our results present evidence for a systematic center-left bias in EC, similar to those observed in many other tasks, mostly involving spatial attention and spatial processing. This biased weighting appears compatible with a limited capacity attentional sampling strategy^16^ affected by how attention is automatically deployed in space. Although we cannot directly rule out this possibility, however, the persistence of this bias under attentional manipulations and the gradual, retinotopically-centered increase from the left to the right side of the visual field, suggest that it may originate early during the encoding of statistical summaries. An interesting question for future studies is whether these anisotropies could vary with exposure time and may be followed by a more extended and uniform distribution of weights.

The exact nature of the anisotropy can be discussed in relation to known hemispheric asymmetries in visual processing. The lateralization and right-hemisphere dominance of functions like salience detection^44^ and global processing^46^, for instance, may lead to the unbalanced weighting of salient stimuli that appear abruptly (as the ensembles in our paradigm) and to global estimates that rely more on information on the left side. Hence, spatially distributed stimuli may trigger the selective activation of the right hemisphere, in turn, generating an anisotropic and leftward biased gradient of weights for summary statistics. More generally, it can be concluded that the right hemisphere has a fundamental role in recognizing the immediate gist of a scene^5^, being specialized in the processing of its defining components, including global shapes^46^, low-spatial frequency content^34^ and, as suggested by our results, summary statistics.

## General methods

### Participants

Eighty-eight participants (Experiment 1: 32, Experiment 2: 27, Experiment 3: 29; 71 females; age range: 18–30) from the University of Fribourg participated in the study for course credits. All participants had normal or corrected-to-normal vision and were naïve as to the purpose of the experiments. Visual acuity was assessed using the Freiburg Visual Acuity Test^47^. The study was approved by the local Ethical Committees of the University of Fribourg and carried out under the Declaration of Helsinki. Written informed consent was obtained from each participant before the experiment.

### Apparatus

Stimuli were presented on a Philips 202P7 CRT (1,600 × 1,200 pixels, 85 Hz) and were generated with a set of custom-made programs written in MATLAB (R2017b) and the Psychophysics Toolbox 3.8, running on Windows-based machines. All experiments were performed in a dimly lit room, and participants sat at a distance of 70 cm away from the computer screen, with their head positioned on a chin rest. All stimuli were presented on a gray background.

### Stimuli

In Experiment 1, stimuli were ensembles of light-gray disks arranged on a 5 × 5 square grid (see Fig. 1A). We used four logarithmically spaced grid sizes (8 × 8°, 11.53 × 11.53°, 16.64 × 16.64°, and 24 × 24°) to present ensembles at four different levels of dispersion (Dispersion levels: 2.3, 3.3, 4.8, 6.9). Disks were centered on each cell of the grid (sides of each cell: 1.6, 2.3, 3.3, 4.8°) with a random horizontal and vertical jitter (ranging from 1 to 40 pixels). To calculate dispersion, we used a measure proportional to the root-mean-square of the distance of each disk from the mean location^48^. Disks at adjacent positions did not overlap. The average size of each ensemble (e.g., the average diameter) was determined starting from four logarithmically spaced seeds (0.75, 0.85, 0.96, 1.10°). The individual sizes of the twenty-five disks were equally spaced on a log scale ranging from ± 0.4° around one of the seeds ^1^. Seeds were randomly selected on each trial and perturbed with a small variation (drawn from a uniform noise distribution with range 0–0.1°). The ensemble grids were always positioned around the center of the screen. Different dispersion levels were interleaved across trials.

Stimuli and task in Experiment 2 (Fig. 1B) were identical to Experiment 1, with the exception that a single grid size was used (12 × 12°) and the ensembles were randomly presented at three different locations, with their center located at either − 6° (left), 0° (Center) or 6° (right) off fixation. In Experiment 3 (Fig. 1C), the display contained two adjacent ensembles of 4 × 4 disks (grid size of 14 × 7° each), grouped by two rectangular frames of a different color (green and red). The two ensembles were centered at either − 12° and − 4° (left side) or 4° and 12° (right side) on different trials.

### Procedure

An example of a trial sequence in Experiment 1 is presented in Fig. 1A. Each trial started with a green fixation cross presented for 720 ms and followed by the ensemble of disks (200 ms). After a blank interval (250 ms), a dark-gray response disk appeared at the center of the screen with a random size (ranging from 0.5° to 8°), and participants were asked to adjust the size to the perceived average diameter of the ensemble. The adjustment response was performed by moving the computer mouse in the upward (increase) or downward (decrease) directions and then clicking the left button to confirm the response. After a variable interval (500–800 ms), a new trial started. The experiment consisted of four blocks of 130 trials each, for a total of 520 trials. The number of ensembles for each dispersion condition was balanced across trials (130 per condition).

In Experiment 2, the sequence and duration of events were identical to those in Experiment 1 and the experiment consisted of four blocks of 120 trials each, for a total of 480 trials (160 trials for each of the three ensemble locations). In Experiment 3, each trial started with a written visual cue (IN or OUT, Fig. 1C) presented either in green or red for 1000 ms, which indicated the position (internal or external) of the relevant ensemble. After a blue fixation cross (720 ms), the two ensembles appeared either on the left or right of fixation, with the green and red surrounding frames serving as spatial cues for the relevant (attended) and irrelevant (non-attended) ensemble. The color assigned to relevant and irrelevant ensembles was fixed for each participant but counterbalanced across participants. To prevent disks from crossing the frames, their range of variation was reduced to ± 0.3° around the average seeds. All other aspects were the same as in Experiment 1–2. Experiment 3 consisted of four blocks of 112 trials for a total of 448 trials, equally distributed between the side of the ensembles (left or right) and the position of the relevant disks (internal or external).

Before each experiment, participants underwent a brief practice session (~ 20 trials). The experiments lasted approximately one hour each.

### Analysis

Before statistical analysis, trials containing absolute adjustment errors larger than 3.5 standard deviations from the mean and reaction times faster than 200 ms or slower than 10 s were removed from subsequent analyses. The average absolute error and the adjustment times for each experiment and condition are reported in Table 1, along with the proportion of outlier trials excluded. In all experiments, we found no difference in performance across conditions (repeated measures ANOVA on absolute error and adjustment times, all *p* > 0.05).

**Table 1:** Summary of the performance in size adjustment tasks.

Experiment 1				
Overall				
Absolute error (°)	0.284 ± 0.102			
Adjustment times (s)	1.917 ± 0.483			
Proportion outliers	0.014 ± 0.026			

Dispersion levels	2.3°	3.3°	4.8°	6.9°
Absolute error (°)	0.283 ± 0.118	0.283 ± 0.109	0.281 ± 0.094	0.289 ± 0.100
Adjustment times (s)	1.917 ± 0.505	1.925 ± 0.482	1.911 ± 0.498	1.916 ± 0.466

Experiment 2				
Overall				
Absolute error (°)	0.291 ± 0.174			
Adjustment times (s)	2.120 ± 0.739			
Proportion outliers	0.026 ± 0.068			

Ensemble location	Left	Center	Right	
Absolute error (°)	0.291 ± 0.174	0.292 ± 0.172	0.290 ± 0.178	
Adjustment times (s)	2.101 ± 0.732	2.150 ± 0.744	2.111 ± 0.750	

Experiment 3				
Overall				
Absolute error (°)	0.391 ± 0.184			
Adjustment times (s)	1.676 ± 0.550			
Proportion outliers	0.023 ± 0.028			

Target ensemble	External left	Internal left	Internal right	External right
Absolute error (°)	0.374 ± 0.182	0.410 ± 0.203	0.394 ± 0.182	0.384 ± 0.188
Adjustment times (s)	1.686 ± 0.537	1.663 ± 0.558	1.655 ± 0.538	1.699 ± 0.584

For each experiment and condition, the overall absolute adjustment error is reported in degrees (°) and the average adjustment times in seconds (s).

Spatial weighted average maps (SWMs), describing the contribution of each disk to the reported average, were obtained by estimating a weighted average model^35,49^ of the form:

1 $$y_{j} = a + \mathop \sum \limits_{i = 1}^{n} w_{i} x_{ij}$$

where $y$ is the adjustment response on trial $j$, $a$ is a constant accounting for systematic biases in over- or under-estimating the average, $x$ is the size of each disk $i$ in the ensemble of $n$ disks ($n = 25$), and $w$ is the vector of spatial weights that map the size of each disk at each location onto the reported average. The vector of estimated weights $w$ was then reshaped into the original 5 × 5 matrix with the actual spatial ordering of disks in the ensemble (Fig. 1D). Heatmaps were generated by filling a matrix of the same size of the screen with the estimated $w$ at the corresponding disk locations (including jitters). For the graphical purpose, the resulting images were smoothed with a two-dimensional Gaussian filtering kernel whose standard deviation was increased by a multiplicative factor (15) of the dispersion value.

In Experiment 1, maps were derived for each participant and dispersion condition separately and their anisotropy was assessed through permutation statistic. A measure of deviation ($\delta$) of the observed weights from a theoretical uniform map (equal weights at all locations) was computed as:

2 $$\delta = \mathop \sum \limits_{i = 1}^{n} \left( {\tilde{w}_{i} - 1/n} \right)^{2}$$

where $\tilde{w}$ are the group-averaged estimated weights for each SWM. Statistical significance was assessed by comparing the observed $\delta$ to a surrogate null distribution. Surrogate $\delta$ were obtained by estimating $w$ (Eq. 1) 5000 times, shuffling the location of the disks ($x$) on each trial. This procedure preserved the true average size on each trial while removing any effect of the spatial organization of disks in the ensemble. To evaluate differences in the spatial distribution of weights across dispersion levels, we performed a permutation *F* statistic. Weights estimated at each location and for each subject were used in a repeated-measures ANOVA with dispersion levels as the main factor. The resulting *F* values were then compared to a null distribution of surrogate *F* values, obtained by shuffling labels of Dispersion levels across participants 5000 times. Statistical significance was computed as the proportion (*p*) of surrogate *F* values below the observed ones, adjusted for a false discovery rate of 5% ^50^. To estimate asymmetries in the contribution of disks presented in the upper/lower or left/right side of the ensemble, as well as to compare the weights of central with lateral disks, we separately averaged columns and rows of the estimated SWM maps for each participant. We then compared weights between specular locations in the upper and lower field (after column averaging, e.g., uppermost row vs. lowermost row) and between the left and right field (after row averaging, e.g., leftmost column vs. rightmost column) using paired t-tests. Similarly, weights on the central part of the ensemble were directly compared to weights in the upper/lower field (e.g., the central row vs. the average of all other rows, after column averaging) and in the left/right field (e.g., the central column vs. the average of all other columns, after row averaging).

In Experiment 2, we directly compared the averaged weights in the two leftmost columns with those in the two rightmost columns, separately for ensembles presented at different locations. This provided a measure of left-side bias for each ensemble (e.g., weights in the two leftmost columns minus weights in the two rightmost columns) that we submitted to a linear regression model, in order to evaluate increases in the left-side bias as a function of the ensemble location. Similarly, in Experiment 3 we derived a measure of the left-side bias by comparing averaged weights for the two leftmost and rightmost columns. This was done for both relevant and irrelevant ensembles, for each combination of the side of the ensembles (left or right) and the position of the relevant disks (internal or external). Following the analysis of Experiment 2, the measure of left-side bias for the relevant ensembles in Experiment 3 was then submitted to a linear regression model, to evaluate increases in the bias as a function of the ensemble location.

## Data Availability

The code used in this study, and the analysed dataset are available at https://zenodo.org/record/4771901.
