# Author Correction: The anisotropic field of ensemble coding

**DOI:** 10.1038/s41598-021-95954-z

**Published:** 2021-08-11

**Authors:** David Pascucci, Nadia Ruethemann, Gijs Plomp

**Affiliations:** 1 Department of Psychology, University of Fribourg, Fribourg, Switzerland; 2 Laboratory of Psychophysics, Ecole Polytechnique Fédérale de Lausanne (EPFL), Lausanne, Switzerland

Correction to: *Scientific Reports* 10.1038/s41598-021-87620-1, published online 15 April 2021

The original version of this Article contained errors. A Data and Code Availability section was omitted. It has now been included and states:

**Data and Code Availability**

The code used in this study, and the analysed dataset are available at https://zenodo.org/record/4771901.

Additionally, one of the *p*-values for Experiment 1 was incorrectly calculated and is now updated. In the Results, subheading ‘Experiment 1’,

“This revealed a bias toward the center and left-hand side of the ensemble (center vs. lower and upper locations: *t*(31) = 4.17, *p* < 0.001, *d*′  = 0.74, Fig. 2B; center vs. left-side and right-side locations: *t*(31) = 2.12, *p* = 0.04, *d*′ = 0.37; leftmost column vs. rightmost column: *t*(31) = 3.25, *p* < 0.001, *d*′ = 0.57; all other *p* > 0.05; paired t-tests, Fig. 2C).”

now reads:

“This revealed a bias toward the center and left-hand side of the ensemble (center vs. lower and upper locations: *t*(31) = 4.17, *p* < 0.001, *d*′ = 0.74, Fig. 2B; center vs. left-side and right-side locations: *t*(31) = 2.12, *p* = 0.04, *d*′ = 0.37; leftmost column vs. rightmost column: *t*(31) = 3.25, *p* = 0.003, *d*′ = 0.57; all other *p* > 0.05; paired t-tests, Fig. 2C).”

The Article has now been corrected.

